# COGNITIVE IMPAIRMENT, FRAILTY, AND GRIP FORCE GENERATION IN PEOPLE LIVING WITH HIV

**DOI:** 10.1093/geroni/igad104.1685

**Published:** 2023-12-21

**Authors:** Kaitlyn Dillon, Bonnie Levin, Roger McIntosh

**Affiliations:** University of Miami, Miami, Florida, United States; University of Miami miller school of medicine, Miami, Florida, United States; University of Miami, Miami, Florida, United States

## Abstract

People living with HIV (PLWH) face an earlier onset and higher rates of geriatric syndromes, including frailty and cognitive impairment. Grip force, an index of frailty, is sensitive to cognitive decline, central nervous system integrity, and may independently predict adverse health outcomes. The current study evaluates frailty, weakness, and cognitive impairment, as well as the neural correlates of grip force as they predict cognitive impairment. PLWH aged 55+ (N = 26; M = 61.9, SD = 4.7; 53.8% female; 76.9% Black) completed an fMRI task in which they were asked to generate 0%, 20%, 50%, and 80% of their maximum force, a neuropsychological battery, and a physical assessment. Cognitive impairment was indexed using the Global Deficit Score (GDS). Per Fried’s Frailty Phenotype, 3.8% of the sample was robust, 57.7% was pre-frail, and 38.5% was frail. Impairment in memory significantly predicted GDS, controlling for age (B = 0.3, t = 4.9, p < .001). Robust frailty status significantly predicted GDS, controlling for age (B = -1.1, t = -2.4, p < .05). Age was also a significant predictor of GDS, controlling for frailty status (B = -0.1, t = -2.7, p < .05). Weakness, as indexed by grip strength, predicted frailty (F = 4.5, p < .05). Additional analyses will be conducted to examine activation in brain regions germane to force generation during the fMRI task as a predictor of GDS. To our knowledge, this is the first study to examine neural correlates of grip force generation in PLWH.

